# Direct and Indirect Modulation of T Cells by VEGF-A Counteracted by Anti-Angiogenic Treatment

**DOI:** 10.3389/fimmu.2021.616837

**Published:** 2021-03-29

**Authors:** Morgane Bourhis, Juliette Palle, Isabelle Galy-Fauroux, Magali Terme

**Affiliations:** ^1^ Université de Paris, PARCC, INSERM, Paris, France; ^2^ Department of GI Oncology, AP-HP, Hôpital Européen Georges-Pompidou, Paris, France

**Keywords:** VEGF-A (vascular endothelial growth factor-A), effector T-cells, regulatory T (Treg) cells, tumor, anti-angiogenic therapy, immunosuppression, immune check point inhibitor (ICI)

## Abstract

Vascular endothelial growth factor A is known to play a central role in tumor angiogenesis. Several studies showed that VEGF-A is also an immunosuppressive factor. In tumor-bearing hosts, VEGF-A can modulate immune cells (DC, MDSC, TAM) to induce the accumulation of regulatory T-cells while simultaneously inhibiting T-cell functions. Furthermore, VEGFR-2 expression on activated T-cells and FoxP3^high^ regulatory T-cells also allow a direct effect of VEGF-A. Anti-angiogenic agents targeting VEGF-A/VEGFR contribute to limit tumor-induced immunosuppression. Based on interesting preclinical studies, many clinical trials have been conducted to investigate the efficacy of anti-VEGF-A/VEGFR treatments combined with immune checkpoint blockade leading to the approvement of these associations in different tumor locations. In this review, we focus on the impact of VEGF-A on immune cells especially regulatory and effector T-cells and different therapeutic strategies to restore an antitumor immunity.

## Introduction

Vascular endothelial growth factor A (VEGF-A) is considered to be a critical regulator of angiogenesis, which is the formation of new blood vessels from pre-existing ones, both in physiological and pathological states such as tumor angiogenesis (1). VEGF-A production is regulated by transcriptional factors such as HIF-1 (hypoxia-inducible factor 1) during a hypoxic stress or by oncogenes (2). Its pro-angiogenic activities are mediated by the activation of VEGF receptors (VEGFR-1, VEGFR-2) which can be expressed on endothelial cells, tumor cells and some immune cells (1). However, VEGF-A has a dual function in supporting tumor progression: first, by inducing vessel formation and second, by acting as an immunosuppressive factor (3–5). The immune system has emerged as a pivotal actor in controlling tumor growth. Cytolytic CD8^+^ T lymphocytes, which have been previously activated by mature dendritic cells presenting tumor antigen-derived peptides, can lyse tumor cells. However, different escape mechanisms are developed by the tumor to evade the immune system such as the development of regulatory T cells (Tregs) or the induction of T cell exhaustion (6). Different works have highlighted a direct or an indirect impact of VEGF-A on this T cell-based immunosuppression. This review will summarize these studies and focus on the immunomodulation induced by anti-angiogenic agents.

## Regulatory T-Cells

Tregs play a critical role in immune homeostasis by regulating effector T cell functions. The proportion of Tregs is enhanced in tumor-bearing mice and in cancer patients and is often associated with a poorer overall survival (7). A correlation between VEGF-A in malignant effusions and the accumulation of Tregs has been observed in cancer patients suggesting a potential role of VEGF-A on Tregs (8). A meta-analysis also revealed that VEGF-A expression is positively associated with intratumoral Tregs in hepatocellular carcinoma (HCC) (9). Thus, VEGF-A has been associated with the induction and maintenance of regulatory T-cells in tumor microenvironment in a direct or indirect-dependent manner.

### Indirect Induction of Regulatory T-Cells by VEGF-A

In cancer, Tregs accumulation could occur through different mechanisms, such as expansion of pre-existing Tregs or conversion of conventional CD4^+^T cells into Tregs. Immature dendritic cells (DC) can induce Tregs proliferation in a TGF-β-dependent manner in tumor-bearing rodents (10). An initial study showed that tumor cell lines-derived VEGF-A affects the development of hematopoietic progenitor cells (HPCs) at an early state resulting in impaired DC differentiation and maturation (11). Inhibition of DC differentiation is mediated by VEGFR-2 (12). In mouse models, VEGF-A binding to VEGFR-1 on HPCs blocked the activation of nuclear factor κB (NF-κB) thereby blocking DC maturation (13, 14). In cancer patients, an increased VEGF-A plasma level is correlated with the presence of immature DC in the peripheral blood (15). This team also reported that the decrease of mature DC is associated with an increase of myeloid-derived suppressor cell (MDSC) in the peripheral blood of cancer patients (16). MDSC, especially Gr-1^+^CD11b^+^CD115^+^ (monocytic) MDSC, can also generate tumor specific Tregs in tumor-bearing mice and in cancer patients by secreting IL-10 and TGF-β (17, 18) or by arginase activity (19). VEGF-A has also been involved in MDSC increase in a VEGFR-2-dependent manner in mice (13, 20) and in ovarian cancer patients (21). Activation of JAK2 -STAT3 pathway by VEGF-A facilitates circulating MDSC accumulation (22). Accumulation of VEGFR-2^+^ MDSC in tumors contributes to poor prognosis (21). Thus, VEGF-A can act on both DC maturation and MDSC in tumor-bearing hosts. These myeloid cells producing immunosuppressive factors such as TGF-β or IL-10 could be involved in Tregs accumulation. Furthermore, a correlation has been observed between MDSC decrease and Tregs reduction in metastatic renal cell cancer patients during sunitinib treatment (a tyrosine kinase inhibitor targeting VEGFR) suggesting a link between MDSC and Tregs (23)

### VEGF-A Directly Promotes Regulatory T-Cells Proliferation

Different studies have recently highlighted a population of Tregs expressing VEGFR-2 in tumor-bearing mice and cancer patients (24–26). In a mouse model of colorectal cancer, we observed that a subset of activated/memory Tregs express VEGFR-2 (unlike healthy controls) and that VEGF-A induces Tregs proliferation in a VEGFR-2 dependent manner (25). In humans, Suzuki et al. showed that VEGFR-2 is selectively expressed by human FoxP3^high^ Tregs but not on FoxP3^low^ Tregs and may have stronger suppressive function (24). Effector CD45RA^-^FoxP3^+^CD4^+^ Tregs subset infiltrating the tumors has also been reported to express VEGFR-2 in advanced gastric cancer patients. Furthermore, the ability of VEGF-A to increase Tregs proliferation has been confirmed in this setting (26). VEGFR-2^+^ Tregs in tumor tissues is also associated with clinical outcome since intratumoral FoxP3^+^ VEGFR-2^+^ Tregs, unlike intratumoral FoxP3^+^ VEGFR2^-^ Tregs are significantly correlated with poor overall survival and disease-free survival. It is an independent factor of recurrence and poor survival in colorectal cancer patients suggesting that VEGFR-2^+^ Tregs may be a prognostic biomarker in colorectal cancer (27). In some tumor locations, the prognostic role of tumor-infiltrating Tregs is still controversed. Taking VEGFR-2^+^ Tregs into account and not all Tregs could be more accurate to evaluate patient prognosis. Furthermore, specifically targeting VEGFR-2^+^ Tregs and not all Tregs could be of interest in cancer patients since it could help to restore an efficient anti-tumor response while limiting autoimmune adverse events.

## Effector T-Cells

Disruption of effector T cell infiltration or activation are important mechanisms of tumor-induced immunosuppression. VEGF-A has also been reported to take part in these mechanisms.

### Immunosuppressive State Mediated by VEGF-A Inhibits Effector T-Cells Functions

As we described above, VEGF-A can block DC maturation and increase MDSC accumulation. Therefore, immature DC are not able to efficiently activate T-cells (11). MDSC are also highly efficient at suppressing effector T cells by different mechanisms: L-arginase depletion (28), NO or ROS production (29, 30) and CD40-CD40L ligation (31). Likewise, tumor-associated macrophages (TAM) express PD-L1 which upon binding with PD-1 inhibits TCR signaling leading to an inactivation of T-cells (32). VEGF-A contributes to TAM recruitment; mainly into poorly vascularized tumor areas, exercising a chemoattractant effect *via* VEGFR-1 expression on macrophages surface. Nevertheless, VEGF-A alone is not sufficient to their activation which requires other tumor-produced factors such as IL-4 and IL-10 (33, 34). The up-regulation of these pro-inflammatory cytokines seems to be favored by VEGF-A over-expression.

### Aberrant Tumor Vasculature Mediated by VEGF-A Decreases T-Cell Infiltration of the Tumor

Although tumor angiogenesis driven by pro-angiogenic factors intends to contribute to blood supply to the tumor, the induced vascular network is abnormal. It is characterized by chaotic, immature, disorganized, poorly perfused and permeable blood vessels which are partially mediated by an abnormal level of tumor-secreted VEGF-A and other factors such as TGF-β, PDGF (platelet-derived growth factor) and angiopoietin 2 (35, 36). In many human and mouse solid tumors, the aberrant structure and function of the tumor vasculature generates a barrier to the CD8^+^ T-cell infiltration and contribute to the maintenance of an immunosuppressive tumor microenvironment (37). Deletion of Rgs5-gene (regulator of G-protein signaling 5), which is responsible for the aberrant morphology of blood vessels, induced a vascular normalization and CD8^+^ T-cell infiltration in tumor-bearing mice (38). Several *in vitro* studies have demonstrated that the decrease of T-cell adhesion resulting in a restricted migration is associated with the decrease of intercellular adhesion molecules 1 (ICAM-1) and vascular cell adhesion molecules 1 (VCAM-1) on endothelial cells (39–41). The cooperation of VEGF-A with IL-10 and prostaglandin E2 is also able to induce FasL expression on tumor endothelial cells. In ovarian, colon, bladder, prostate, and renal cancers, FasL^+^ endothelial cells acquire the ability to kill T-cells while allowing FoxP3^+^ Tregs accumulation and infiltration (42). Finally, the down-regulation of adhesion molecules and expression of FasL on tumor endothelial cells mainly induced by VEGF-A are responsible for a decrease of tumor infiltration by T-cells.

### VEGF-A Directly Suppresses T-Cell Functions

In tumor producing elevated levels of VEGF-A, studies revealed that this factor and its receptors have important roles in the aberrant hematopoiesis resulting in defects in immunity (20). Mice exposed to recombinant VEGF at similar concentrations to those observed in patients with advanced cancer develop a thymic atrophy with a reduced number of CD4/CD8 thymocytes (43). These results demonstrate that VEGF-A directly interferes with the thymic development of T-cells from HPCs and can contribute to the immune deficiencies associated with tumors. Studies revealed that VEGF-A directly impacts effector T cells. Indeed, *in vitro* activated T-cells but also tumor-infiltrating T cells express VEGFR-2 (44). In advanced ovarian cancer, VEGF-A directly suppresses T cell proliferation and cytotoxic activity *via* VEGFR-2 (45, 46). Although Basu et al.’ study reported an enhanced IFN-γ and IL-2 production and migratory responses induced by VEGF-A in human CD45RO^+^ CD4^+^ memory T-cells (47), there are growing evidences to support the immunosuppressive role of VEGF-A/VEGFR in T-cells (5) especially on tumor-induced T-cell exhaustion (44, 48). T-cell exhaustion is phenotypically characterized by the co-expression of immune inhibitory receptors called immune checkpoints such as program cell death-1 (PD-1), T-cell immunoglobulin mucin-3 (Tim-3), cytotoxic T-lymphocyte-associated protein (CTLA-4), lymphocyte activation gene 3 (Lag3) in CD8^+^ T-cells and by a gradual loss of function (49). VEGF-A increases PD-1 expression and other immune checkpoints CTLA-4, Tim-3 and Lag-3 on CD8^+^ T-cells but also their co-expression which is related to exhaustion. The VEGFR-2- PLCγ- calcineurin- NFAT pathway is involved in this effect (44). These results have been confirmed by others (48, 50). Recently, a study carried out on patients with microsatellite stable colorectal cancer (MSS CRC) resistant to anti-PD-1 therapies has identified that VEGF-A-dependent upregulation of immune checkpoints involved the TOX transcription factor (50).

**Figure 1:**
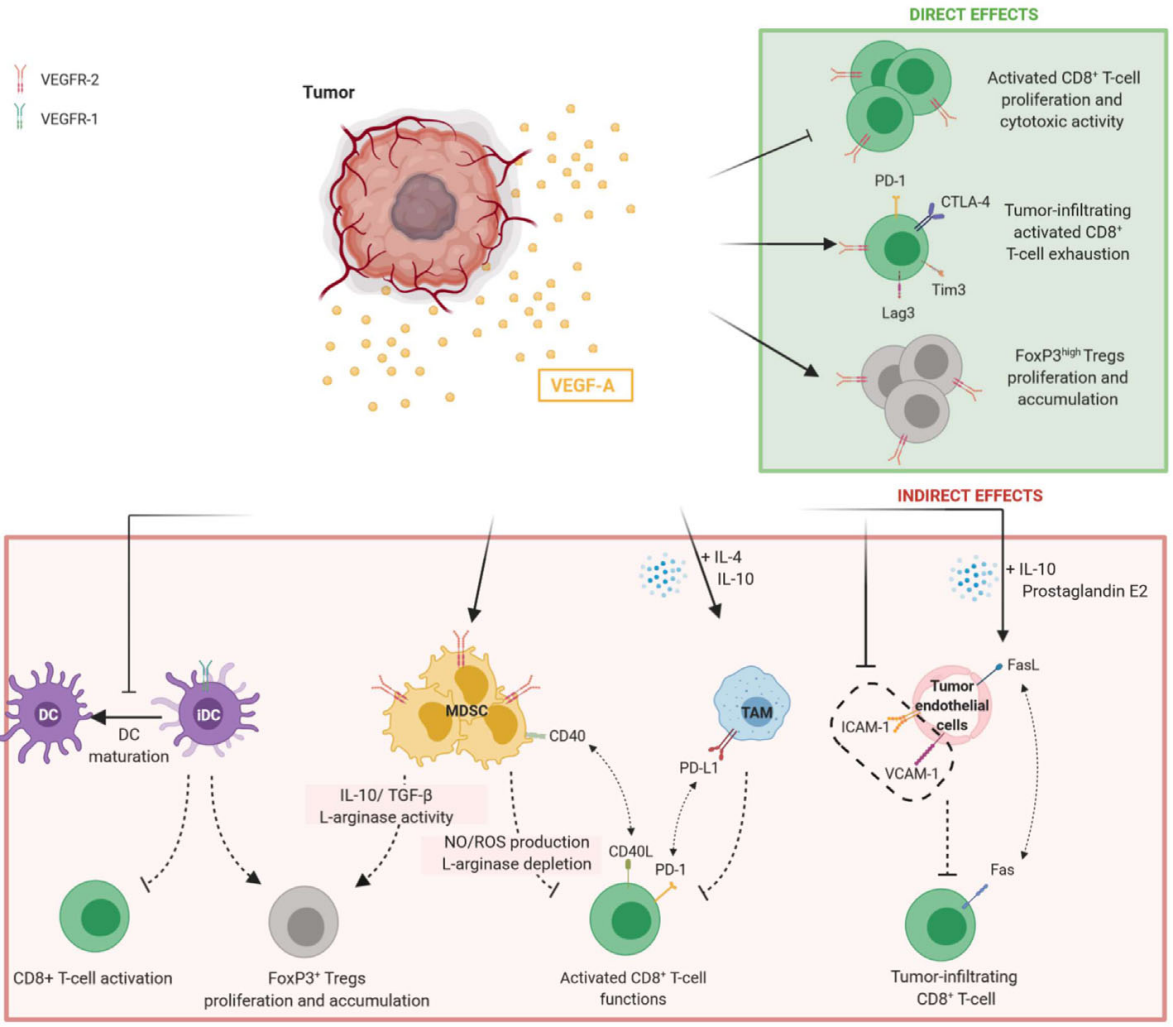
Direct and indirect effects of VEGF-A in promoting immunosuppressive tumor microenvironment. Tumor-secreted VEGF-A induces the inhibition of T-cell functions and proliferation while promoting Tregs accumulation. Both direct and indirect effects of VEGF-A are observed. Direct modulation of T-cell by VEGF-A is mediated by the binding to VEGFR-2 on their surface, whereas the indirect effects of VEGF-A on T-cell results from the modulation of immune cells such as DC, MDSC and TAM expressing VEGFR-1 or VEGFR-2. On tumor endothelial cells, VEGF-A can reduce adhesion molecules expression and induce FasL, preventing tumor-infiltrating CD8^+^ T-cell. DC, dendritic cells; iDC, immature dendritic cells; MDSC, myeloid derived suppressor cells; TAM, tumor-associated macrophages.

In conclusion, VEGF-A acts as an immunosuppressive factor in modulating immune cells. Its effects are summarized in **Figure 1**.

## Anti-Angiogenic Therapies

Based on these properties, the immunomodulatory role of anti-angiogenic (AA) agents targeting VEGF-A/VEGFR in antitumor immunity has been investigated in tumor models and cancer patients (51). These effects are summarized in **Table 1**.

**Table 1:** Modulation of T-cells by anti-angiogenic therapy.

Anti-angiogenic	Target	Effects of anti-angiogenic therapy	
**Tyrosine Kinase Inhibitor**			
Sunitinib	VEGFRs(+ c-Kit, PDGFR,Flt-3)	Decrease the percentage of splenic Tregs^*^	(23)
		Decrease the percentage of circulating Tregs (correlated with reduction of MDSC numbers in TME)^d^	(53–58)
		Suppress the conversion of CD4^+^ CD25^-^ T cells in Tregs^d^	
		Enhance Th1 cytokine response (IFN-g production)^d^	
		Favor CD4^+^ and CD8^+^ T-cells infiltration in the tumor^*^	
		Reduce PD-1 expression of intra-tumoral CD8^+^ T-cells^*^	
Sorafenib	VEGFRs(+ c-Kit PDGFR, Raf-kinases, RET)	Decrease Tregs proportion (correlated with reduction of MDSC numbers in the TME)^d,e,*^	(59–61) (64, 65)
		Enhance effector T-cell functions^e^	
		Reduce PD-1 expression on CD8^+^ T-cells^e,*^	
Axitinib	VEGFR-2	Induce the reversal of T-cell suppression through the suppression of MDSC accumulation^*^	(66–68)
		Increase CD8^+^ T-cells proportion^*^	
		Favor immune cells infiltration in the tumor^*^	
**Monoclonal antibody**			
Bevacizumab	VEGF-A	Decrease the percentage of splenic Tregs^*^ and circulating Tregs^a^	(8, 29, 44)
		Increases CD4, CD8 and CD3 lymphocyte numbers and T-cell proliferation^a^	(69–71)
		Enhance cytokine production of circulating T-cells^a^	
		Enhance cytotoxic T-lymphocytes responses^b^	
		Reduce PD-1 expression on intra-tumoral CD8^+^ T-cells^*^	
		Limit co-expression of inhibitory checkpoint associated with exhaustion^*^	
Ramucirumab	VEGFR-2	Reduce effector Tregs (CD45RA^-^ FoxP3^+^ CD4^+^ Tregs) expressing VEGFR-2^c^	(26)
		Reduce PD-1 expression on CD8^+^ T-cells (only for patients with high frequency of effector Tregs before treatment)^c^	
**Fusion Protein**			
Aflibercept		Enhance CD8^+^ T-cells functions^*^	(72)
		Reduce PD-1 and Tim3 expression of intra-tumoral CD8^+^ T-cells^*^	

a Colorectal cancer patients.

b Non-small cell lung cancer patient.

c Gastric cancer patients.

d Renal cell carcinoma patients.

e Hepatocellular carcinoma patients.

^*^tumor-bearing mice.

### Anti-VEGF-A/VEGFR Therapies Modulate Immune Cells Including T-Cells

During the last decade, different AA molecules have been developed and approved to treat cancer patients. They can be classified in three main classes: (i) small tyrosine kinase inhibitors (TKI) such as sunitinib, sorafenib and axitinib (ii) monoclonal antibodies (mAb) such as bevacizumab (anti-VEGF-A) and ramucirumab (anti-VEGFR-2) (iii) aflibercept which is a fusion protein composed of extracellular domains from VEGFR-1 and VEGFR-2 (52). Whereas TKI target VEGFR pathway (but also other receptors), monoclonal antibodies and fusion proteins directly target circulating pro-angiogenic factors or their receptors present on the cell membrane.

#### Tyrosine Kinase Inhibitors

***Sunitinib.*** Sunitinib is a TKI currently used to treat different types of cancers, in particular metastatic renal cell carcinoma (mRCC) (52). After sunitinib treatment, the percentage of splenic FoxP3^+^ Tregs in mouse models of renal cancer and circulating Tregs in mRCC patients is decreased (23, 53–55). Sunitinib also reduces MDSC numbers which is correlated with Treg decrease in the tumor microenvironment (23, 56) and favors CD4^+^ and CD8^+^ infiltration in the tumor site while reducing PD-1 expression on CD8^+^ T-cells (44, 53). Moreover, sunitinib suppresses the conversion of CD4^+^CD25^-^ naïve T cells in Tregs in mouse tumor models (57). In humans, *in vitro* studies reported a significant improvement of Th1 cytokine response in mRCC patients receiving sunitinib. This effect seems to be linked to a reduction of Tregs (23, 54). In addition, in RCC tumor cells and tumor-associated MDSC, sunitinib inhibits Stat3 activity leading to tumor cell apoptosis and promoting antitumor effect (56).

***Sorafenib.*** As well as sunitinib, sorafenib can reduce Tregs and MDSC proportion in mouse models of liver cancer (58) and in HCC patients (59) or RCC (60). However, sorafenib seems to modulate T-cell functions differently from sunitinib and had no impact on Th1 response (60, 70). An *in vitro* study examining the effects of sorafenib on the proliferation and activation of human peripheral blood T-cells showed that sorafenib targets LCK phosphorylation implicated in the TCR signaling causing the loss of T-cell immune responses (71). Controversially, studies have revealed that this treatment seems to up-regulate the tumor-specific effector T cell functions while PD-1 expression on CD8^+^ T-cells is down-regulated (59, 61, 62). Currently, the effects of sorafenib on T-cell functions remain unclear.

***Axitinib.*** Axitinib is a highly selective VEGFR tyrosine kinase inhibitor and has demonstrated its efficacy in the treatment of advanced RCC (aRCC) (63). In tumor bearing mice, axitinib suppressed MDSC accumulation through the inhibition of Stat3 activity and it was correlated with the reversal of T-cell suppression (64, 65). Indeed, the proportion of CD8^+^ T-cells is increased in a mouse model of renal cancer (64) and an *in vivo* study in a mouse melanoma model showed an increase of tumor-infiltrating immune cells (65).

#### Monoclonal Antibodies

***Bevacizumab.*** Bevacizumab, a humanized anti-VEGF-A monoclonal antibody which directly targets VEGF-A, reduced the proportion of Tregs in tumor-bearing mice and in patients with metastatic colorectal cancer (mCRC) (25, 66). This phenomenon is linked to a decrease of Ki67^+^ expression in Tregs (25). In mCRC patients treated with bevacizumab, Manzoni et al. revealed an increase of CD4, CD8 and CD3 lymphocyte numbers (29), whereas Tsavaris et al. observed a better proliferation and cytokine production of circulating T-cells compared to patients treated with chemotherapy only (67). Moreover, the administration of anti-VEGF-A in tumor-bearing mice decreased PD-1 expression on intratumoral CD8^+^ T-cells and limited the co-expression of inhibitory checkpoints associated with exhaustion (44). Recently, a study conducted on non-small-cell lung cancer (NSCLC) patients revealed that bevacizumab addition to the chemotherapy doublet based on cisplatin and oral etoposide decreased the plasmatic VEGF-A level and improved cytotoxic T-lymphocytes responses (68) while simultaneously restoring DC functions.

***Ramucirumab.*** Ramucirumab is a monoclonal antibody targeting VEGFR-2. In patients with advanced gastric cancers, CD45RA^-^ FoxP3^+^ CD4^+^ effector Tregs cells expressing VEGFR-2 are present in higher frequency in TIL than in PBMC (26). In vitro experiments showed that VEGF-A stimulates their proliferation which can be overcome by ramucirumab. Ramucirumab-containing therapies strongly reduce effector Tregs in tumors of advanced gastric cancer patients. A higher frequency of these effector Tregs in tumors before treatment was associated with an enhanced proportion of partial response and a longer progression-free survival. Since VEGFR-2 is highly expressed by this Tregs subset, it could be envisioned that the proportion of VEGFR-2^+^ effector Tregs in TIL could be a biomarker of favorable clinical response to ramucirumab therapies (26). A decreased expression of PD-1 on CD8^+^ T-cells was also reported after ramucirumab treatment (26).

#### Fusion Protein

***Aflibercept.*** Aflibercept is a VEGFR fusion protein conjugated to Fc portion of human IgG1, also known as VEGF-Trap. Nowadays the impact of VEGF-Trap treatments on T cells remains poorly described in cancer patients but a recent study highlights the improvement of CD8^+^ T cell functionality in a mouse model of glioma. Indeed, they observed a decrease of PD-1 and Tim3 expression on infiltrating CD8^+^ T-cells. Likewise, they found an improvement of DC maturation demonstrated by the increase of co-stimulation molecules expression including CD80, CD86 and MHC II which are required for T-cell activation (69).

### Indirect Impact of Anti-Angiogenic on Immune Cells

An indirect impact of AA treatment on tumor-induced immunosuppression could also be proposed. AA treatments can be responsible of a transient normalization of the vasculature favoring tumor infiltration by immune cells. However, some reports have also indicated a potential enhancement of hypoxia especially during a prolonged AA treatment (72). Hypoxia fuels tumor progression by selecting more malignant cells and also by inducing an immunosuppressive microenvironment. It can lead myeloid cells toward an immunosuppressive phenotype or potentiate regulatory T cell functions (73). Nevertheless, recent studies highlighted that aggravated hypoxia mediated by anti-VEGF-A treatment directly enhances CD8^+^ T-cell functions in an HIF-1α dependent manner (74, 75). This aspect of anti-angiogenic impact needs further investigation.

### Combination of Anti-VEGF-A/VEGFR Treatments With Immunotherapies

To enhance anti-tumor effects, combining AA therapies with immunotherapies such as immune checkpoint blockade (ICB: anti-PD-1, anti-PD-L1, anti-CTLA-4) have raised great interest. In mouse tumor models, VEGF-A/VEGFR-2 and PD-1 blockade induces strong and synergic antitumor responses and limits T-cell exhaustion in VEGF-A-expressing tumor compared to monotherapies in mouse models of MSS colorectal cancer (44, 50). Two studies have been carried out in murine lung cancer models using anti-VEGF/VEGFR combined to anti-PD-L1. They have demonstrated strong anti-tumor effects which are associated with an increase of TIL and T-cell responses (48, 76). The association of anti-VEGFR-2 plus anti-PD-L1 could rescue the PD-1/Tim3 exhaustion T-cell phenotype, while improving overall survival (48). In preclinical murine models, the association of axitinib with ICB (anti-PD-L1 and anti-Tim3 antibodies) resulted in a synergistic therapeutic efficacy (77).

Based on the interesting results from preclinical studies, many clinical trials have been conducted to evaluate combination therapies in cancer patients (78). In 2014, a phase I clinical trial (NCT00790010) investigated the combination of ipilimumab, an anti-CTLA-4 monoclonal antibody, and bevacizumab in 46 patients with a metastatic melanoma (79). The authors observed an upregulation of VCAM-1 and other adhesion molecules on intratumoral endothelial cells leading to endothelial cell activation. Furthermore, the trafficking of CD8^+^ T-cells across tumor vasculature was enhanced. When combined to ipilimumab, bevacizumab seems to influence tumor vasculature morphology and immune responses (79). Although antitumor response efficacy has been demonstrated, important immune-related adverse events are induced. Anti-CTLA-4 antibodies are known to generate autoimmune diseases (80). Meta-analysis revealed that about 70% of patients treated with ipilimumab including 25% of high-grade adverse events develop skin rashes, gastrointestinal dysfunctions such as colitis or less frequently endocrine dysfunctions (81, 82). When ipilimumab was combined to bevacizumab, 29.3% of patients experienced grade 3-4 adverse events. Inflammatory events were higher than expected with ipilimumab alone, but remained manageable. Interestingly, the incidence of colitis was not increased (79). High grade adverse events are less common for anti-PD-1/PD-L1 antibodies alone compared to ipilimumab. A study conducted in a small cohort of mRCC patients explored the effects of an anti-PD-L1 (atezolizumab) plus bevacizumab (NCT01633970) (83). Similar findings were highlighted including an improved migration of antigen-specific T-cells and an increase of cytokines and chemokines production in particular CX3CL1 involved in T-cell trafficking (83). Therapies combining AA agents plus ICB have shown their efficiency in phase III clinical trials (84) and have been recently approved in different locations (i) atezolizumab (anti-PD-L1) and bevacizumab with chemotherapy in NSCLC (85) (ii) atezolizumab and bevacizumab in unresectable HCC (86) (iii) pembrolizumab (anti-PD-1) plus lenvatinib (TKI) in advanced endometrial cancer (87) (iv) pembrolizumab (anti-PD-1) plus axitinib in RCC (88) and v) axitinib plus avelumab (an anti-PD-L1) in RCC (89). No major increases of adverse events were reported with these combinations compared to each agent alone. Results of these clinical trials are summarized in **Table 2**.

**Table 2:** Current clinical trials of anti-angiogenic therapies combined with immunotherapies.

Anti-angiogenic	Immunotherapy	Registration number	Phase	Cancer Location	Clinical efficacy	Correlatives
**Tyrosine Kinase Inhibitor**						
Axitinib	Avelumab^2^	NCT02684006	III	Advanced RCC	OS 11.6 monthsPFS 13.8 months	As first-line treatment, improved PFS among patients with PD- L1-positive tumors (89)
	Pembrolizumab^3^	NTC02853331	III	Advanced RCC	OS 89.9%*PFS 15.1 months	(88)
Lenvatinib	Pembrolizumab^3^	NTC03517449	III	Advanced EC		(87)
**Monoclonal antibody**						
Bevacizumab	Ipilimumab^1^	NCT00790010	I	Metastatic melanoma	OS 25.1 months	Enhance the trafficking of CD8^+^T-cells across tumor vasculature (79)
	Atezolizumab^2^	NCT01633970	Ib	mRCC		Improve migration of T-cellsIncrease cytokine and chemokines production (83)
		NTC03434379	III	Unrestable HCC	OS 67.2%*PFS 6.8 months	(86)
	Atezolizumab^2^+ chemotherapy	NTC02366143	III	NSCLC	OS 13.3 months	Improved OS in the subgroup of patient with baseline liver metastasis (85)

^1^anti-CTLA-4 antibody, ^2^anti-PD-L1 antibody, ^3^anti-PD-1 antibody; *at 12 months. (m)RCC, (metastatic) renal cell carcinoma; HCC, hepatocellular carcinoma; NSCLC, non-small cell lung cancer; EC, endometrial cancer; OS, overall survival; PFS, progression-free survival.

### Perspectives for a Better Use of Anti-Angiogenic Agents

The need to find approaches enabling the induction and activation of immune response against cancer remains considerable and in this context, novel approaches are important to consider. A better understanding of the immunomodulatory roles of pro-angiogenic factors produced in tumor-bearing hosts could help to develop new therapeutic strategies or combinations. One of the main issues encountered with AA agents in the development of resistance mechanisms including activation of alternative pro-angiogenic pathways such as Angiopoietin 2 (Ang2)/Tie2, HGF/c-Met or PlGF (90–92). High levels of Ang2 are associated with unfavorable responses to bevacizumab-containing therapies in patients suffering from colorectal cancers and metastatic melanoma treated with ICB (93). Different AA molecules are currently used to treat cancer patients, but the impact of these different treatments on anti-tumor immunity remains unclear. In some tumor locations, combining AA molecules with ICB allowed to improve the outcome of cancer patients, leading to the approval of different anti-angiogenic/ICB combinations by the FDA. However, many questions remain unanswered such as which tumors are sensitive to these associations, or if targeting alternative pro-angiogenic pathways in combination with ICB could also provide interesting anti-tumor effects. Recently, a study has shown in mouse tumor models that the concomitant blocking of VEGF-A and Ang2 with a bi-specific antibody stimulated antitumor immune responses compared with single-agent therapies and could increase the effectiveness of ICB such as anti-PD-1 antibody (94). Currently, multiple clinical trials are being conducted to assess dual inhibition of VEGF-A and Ang2 in patients with cancer and to further investigate mechanisms involved in alternative pro-angiogenic pathways (95).

## Author Contributions

MB and MT designed and wrote the main manuscript. JP and IG-F critically reviewed the manuscript. All authors contributed to the article and approved the submitted version.

## Funding

JP received financial support from ITMO Cancer AVIESAN (Alliance Nationale pour les Sciences de la Vie et de la Santé, National Alliance for Life Sciences & Health) within the framework of the Cancer Plan. MB was supported by Association pour la Recherche contre le Cancer. This work has been supported by Cancéropôle Ile de France/INCa (2017-1-EMERG-74-INSERM-5-1), SIRIC CARPEM, Labex Immuno-oncology and Ligue contre le Cancer.

## Conflict of Interest

MT reports personal fees from Roche and Gilead and research grant from Sanofi outside the submitted work.

The remaining authors declare that the research was conducted in the absence of any commercial or financial relationships that could be construed as a potential conflict of interest.
